# A Comparison of Flow Cytometry-based versus ImmunoSpot- or Supernatant-based Detection of SARS-CoV-2 Spike-specific Memory B Cells in Peripheral Blood

**DOI:** 10.3390/vaccines14010020

**Published:** 2025-12-24

**Authors:** Georgia Stylianou, Sharon Cookson, Justin T. Nassif, Greg A. Kirchenbaum, Paul V. Lehmann, Stephen M. Todryk

**Affiliations:** 1 Faculty of Science and Environment, School of Geography and Natural Sciences, Northumbria University, Newcastle upon Tyne NE1 8ST, UK; georgia3.stylianou@northumbria.ac.uk (G.S.); greg.kirchenbaum@immunospot.com (G.A.K.); 2 Translational & Clinical Research Institute, Newcastle University, Newcastle upon Tyne NE1 7RU, UK; 3 Research & Development, Cellular Technology Limited, Shaker Heights, OH 44122, USApaul.lehmann@immunospot.com (P.V.L.)

**Keywords:** SARS-CoV-2, ELISPOT, FluoroSpot, ImmunoSpot, memory B cell measurement, probe-based flow cytometry, antibody, ELISA, immune monitoring

## Abstract

Background: Memory B cells (B_mem_) facilitate the generation of renewed and rapid antigen-specific antibody responses long after the initial antigen exposure, at a time when circulating serum antibodies may have declined. As the generation and/or recruitment of B_mem_ is at the core of most vaccination strategies, the assessment of antigen-specific B_mem_ is highly informative for forecasting and profiling the elicited B cell immune response. Methods: The two prevalent techniques used to detect antigen-specific B_mem_ cells at single-cell resolution are probe-based flow cytometry and B cell ImmunoSpot, while the measurement of B cell-derived antibodies in culture supernatants of stimulated B cells offers a semi-quantitative alternative. To the best of our knowledge, a direct side-by-side comparison of these assay systems has not yet been reported using the same starting PBMC material in a blinded fashion to test all three assays simultaneously. Results: These three assay systems were run in parallel to detect SARS-CoV-2 Wuhan-1 strain Spike-specific IgG^+^ B_mem_ in peripheral blood mononuclear cell (PBMC) samples obtained from well-defined cohorts comprising pre-COVID-19 era “naïve” individuals (negative controls), individuals shortly after recovery from a PCR-verified SARS-CoV-2 infection (positive controls), and a cohort of donor PBMCs isolated in 2024 (the experimental group). Each assay was able to discern Spike-exposed individuals from naïve , with ImmunoSpot suggesting superior sensitivity and specificity. ImmunoSpot and flow cytometry results were closely correlated. Conclusions: The study demonstrates that all three assays are suited for the detection of specific B_mem_ in antigen-primed individuals when such B_mem_ occur in the mid- to high-frequency range, and that they broadly concur. Strengths and weaknesses of the three test systems are discussed.

## 1. Introduction

Immune monitoring aims to measure whether an immune response has been engaged, its quality and magnitude, as well as to further understand the mechanisms involved. Such is required for optimizing diagnostic, prognostic, and therapeutic management of any immune-mediated conditions, including post-infection- or vaccine-induced immunity against microorganisms, allergic and hypersensitivity reactions, the rejection of transplants, elimination of genetically modified and cancerous cells, and a multitude of autoimmune diseases [1,2,3,4].

Measurements of specific serum antibodies (produced by the plasma cell progeny of B cells) remain the prevalent means of immune diagnosis. However, antibodies in circulation are just one effector arm of the adaptive immune system, alongside that mediated by T cells. As antibodies often either do not even play the central role in the immune reaction of interest (e.g., against intracellular pathogens, cancerous cells, and in several autoimmune diseases) or are just one of the contributing defense mechanisms (e.g., immunity to most infectious agents), recently, increasing efforts have been invested into improving T cell immune diagnostics [5,6,7,8,9,10,11,12]. Moreover, it has become clear in recent years that serum antibodies often do not provide sufficient insights into B-cell-mediated humoral defense [13,14].

During immune responses, B cells clonally expand and differentiate either into antibody-producing plasma cells or B_mem_ (reviewed in [15,16]). The instructive signals for B cell maturation into either of these sub-lineages are different, and so can be the long-term fate of the two [17]. While both cell types are potentially long-lived, plasma cells’ survival depends on competition for limited niches in the bone marrow, whereas B_mem_ do not have such limitations [18,19]. Moreover, with a half-life of approximately 3 weeks, serum antibody molecules are relatively short-lived in vivo, and therefore their continued presence depends on constant replenishment by plasma cells [20,21]. The recent COVID-19 pandemic and mRNA vaccination efforts revealed how fast serum antibody levels can decline [22,23,24,25].

Secreted antibodies and B_mem_ also have fundamentally different roles in contributing to host defense [17,19]. Existing serum antibodies provide the first line of protection, enabling, by instant binding to the antigen, its elimination and the prevention of (re-) infection. When this “first wall of humoral immunity” is insufficient, it is up to B_mem_ to provide “the second wall” by re-engaging in a secondary-type immune response that will give rise to terminally differentiated plasma cells (and therefore antibodies) and more B_mem_ [15,16,17]. While B_mem_ cannot prevent (re-)infection, their somewhat delayed but vigorous engagement can still critically contribute to host defense through curtailing pathogen dissemination and aiding in its clearance. Measuring serum antibodies, therefore, provides only insights into a transient and frequently rapidly fading memory of past immune responses, whereas monitoring of B_mem_ permits the prediction of the host’s potential to generate a secondary-type antibody response (reviewed in [26]).

Monitoring antigen-specific B_mem_ is challenging because these cells represent, even after the strongest of immune responses, only a small fraction of all B cells recirculating in the blood; they generally occur in low to very low frequencies among peripheral blood mononuclear cells (PBMCs) [14,27,28]. Although detecting antigen-specific B_mem_ is clearly a challenge for any technology, several methods have emerged as candidates to measure this fundamental immune parameter [29,30]. The classic approaches of growing individual B cell clones/hybrids are limited by the high labor requirements and low throughput [31]. Single-cell paired *IgH*/*IgL* sequencing is reliable only if the antibodies are recombinantly expressed and tested for their actual antigen-binding properties (as only a fraction of the B cells identified may actually be antigen specific)—an effort again too demanding for comprehensive immune monitoring on sizeable donor cohorts [32]. In this communication, we compare two cellular methods of B_mem_ detection that are scalable and thus have promise to be suitable for the immune monitoring routine. One is staining antigen-specific B cells with fluorochrome-labeled antigen probes followed by their detection via flow cytometric analysis [33]. The other one is ELISPOT/FluoroSpot (collectively called ImmunoSpot) [26]. These two single-cell analysis-based methods were compared to the detection of specific antibodies in supernatants of polyclonally stimulated PBMCs [34]. While each of these assays has been used for B_mem_ detection independently [35], to the best of our knowledge, a systematic side-by-side comparison of their respective lower limits of detection (LLoD) and diagnostic specificity has not been published so far.

In the past, there has never been a blinded side-by-side comparison of the three assays tested in parallel using the same starting material. The SARS-CoV-2 pandemic offered the perfect opportunity to carry out such a side-by-side comparison of probe-based flow cytometry, ImmunoSpot, and the measurement of B-cell-derived antibodies in culture supernatants of stimulated B cells. Prior to November 2019, individuals had not been exposed to the virus and, therefore, could be considered a naïve population, while those from 2024 would be assumed to have been vaccinated and/or infected with the virus. The data presented here were generated to fill this gap, taking advantage of the unique scenario the SARS-CoV-2 pandemic created and highlighting the strengths and weaknesses of the respective techniques in the context of detecting underlying B_mem_ reactivity for the SARS-CoV-2 Spike protein.

## 2. Materials and Methods

### 2.1. Human Donors

Peripheral blood mononuclear cells (PBMCs) originating from CTL’s ePBMC^®^ library (CTL, Shaker Heights, OH, USA) were obtained from IRB-consented healthy adults by leukapheresis at FDA-registered collection centers and then were sold to CTL, identifying donors by code only while concealing the subjects’ identities. PBMCs were subsequently cryopreserved according to previously described protocols [36] and were stored in liquid nitrogen for long-term storage until testing. The PBMC samples (*n* = 36) were shipped to Northumbria University in a liquid nitrogen cryo-shipper in preconfigured storage boxes and could only be identified by the experimenting lab using the unique CTL identifying code to maintain blinding of the experimenter. Following receipt of the cryopreserved PBMC samples, they were immediately transferred into a liquid nitrogen cryostorage tank until testing.

**Figure 1:**
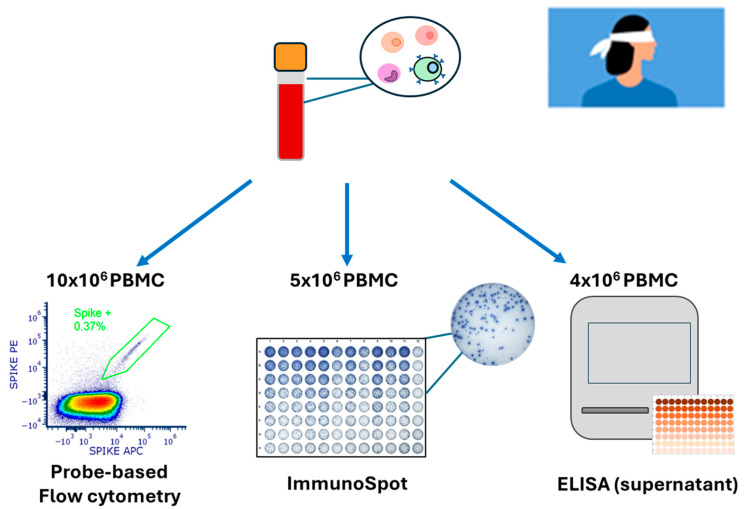
Overview of the experimental workflow. Cryopreserved peripheral blood mononuclear cells (PBMCs) were used for all three assays. (**Left**) Probe-based flow cytometry was performed to identify SARS-CoV-2 Spike-specific B cells using fluorescently labeled Spike antigen (10 × 10^6^ PBMCs were allocated for this assay). (**Middle**) ImmunoSpot^®^ assays were performed using donor PBMCs following 5 days of in vitro polyclonal stimulation, and individual spot-forming units (SFUs) were visualized to measure Spike-specific ASCs (5 × 10^6^ PBMCs were allocated to the assay). (**Right**) Supernatants generated by culturing polyclonally-stimulated PBMCs for 11 days were measured by ELISA to quantify Spike-specific IgG titers. Collectively, these three assays enabled assessment of Spike-specific B cell memory (B_mem_) in cryopreserved PBMC samples originating from the study participants.

The PBMC samples detailed in this study belonged to three separate cohorts (Table 1 and Figure 1). The pre-COVID-19 cohort comprised (*n* = 12) PBMC samples that were cryopreserved before the 1st of November 2019 and originated from subjects inferred to be immunologically naïve to the SARS-CoV-2 virus since the first case of SARS-CoV-2 infection in the United States was not reported until January 2020 [37]. The convalescent cohort comprised (*n* = 12) PBMC samples that were collected from April to October 2020 from subjects that recently recovered from a PCR-verified SARS-CoV-2 infection and prior to the availability of COVID-19 vaccines. Early SARS-CoV-2 variants were already in circulation by April–May 2020. However, these variants were genetically close to the Wuhan-Hu-1 (WH1) strain, and published data demonstrate that they retain strong cross-reactivity with the prototype vaccine strain against the Spike protein. Lastly, (*n* = 12) PBMC samples collected in the year 2024, at which point the majority of the general population in the United States had either previously received one or more COVID-19 vaccine inoculations and/or were infected with the SARS-CoV-2 virus and hence would be expected to have generated immune memory to Spiken, comprised the post-COVID-19, 2024 cohort. Notably, all PBMCs were tested in a blinded fashion (Figure 1), in which the identity of the individual samples and which cohort they belonged to was unknown to the experimenting lab at the time of testing.

### 2.2. Recombinant Proteins

A full-length SARS-CoV-2 Spike protein representing the Wuhan-1 (WH1) strain with a genetically encoded Avi-tag sequence to ensure site-specific biotinylation was purchased from Sino Biological (Beijing, China) and used to generate tetrameric antigen probe complexes for the flow cytometry experiments. Alternatively, for ImmunoSpot assays, an analogous WH1 Spike protein with a genetically encoded C-terminal histidine (His) tag [38] was acquired from the Center for Vaccines and Immunology (CVI) (University of Georgia, Athens, GA, USA).

### 2.3. Polyclonal B Cell Stimulation

Cryopreserved PBMCs were thawed according to previously described methods [36] and resuspended in complete (c) RPMI medium composed of RPMI 1640 (Gibco, Thermo Fisher Scientific, Paisley, UK) supplemented with 10% fetal bovine serum, 100 U/mL penicillin, 100 U/mL streptomycin, 2 mM L-Glutamine, 1 mM sodium pyruvate, 8 mM HEPES (Gibco), and 50 µM β-mercaptoethanol (all from Gibco). A total of 5 × 10^6^ PBMCs were subjected to in vitro polyclonal stimulation in 6-well plates at a final density of 2 × 10^6^ cells/mL in cRPMI supplemented with B-Poly-S ™ (CTL), comprising the TLR7/8 agonist R848 and recombinant human interleukin-2. Culture plates were incubated at 37 °C, 5% CO_2_ for 5 days, during which time memory B cells (B_mem_) underwent proliferation and terminal differentiation into antibody-secreting cells (ASCs). After the 5 days of in vitro culture, the polyclonally stimulated PBMCs were harvested, counted, and then plated into B-cell ImmunoSpot^®^ assays as detailed below. Alternatively, an independent culture of 4 × 10^6^ PBMCs was set up under identical conditions (with B-Poly-S), and the antibody-containing supernatants were collected after 11 days for testing in ELISA for Spike-specific IgG.

### 2.4. ELISA

Detection of Spike-specific IgG in plasma samples was performed according to previously described methods with minor modifications [14]. Briefly, ELISA plates (Immulon^®^ 4HBX, flat-bottom, Thermo Fisher Scientific) were coated with Spike antigen at 2 μg/mL overnight at 4 °C. The plates were decanted and blocked with ELISA blocking buffer containing 2% (*w*/*v*) bovine serum albumin (BSA) (Sigma-Aldrich) in PBS with 0.1% (*v*/*v*) Tween20 (PBS-T) for 1 h at room temperature. Serially diluted plasma samples were added and incubated overnight at 4 °C. The following day, plates were washed with PBS, and horseradish peroxidase (HRP)-conjugated anti-human IgG detection reagent (from CTL) was added with incubation for 1 h at room temperature. After washing, TMB chromogen solution (Thermo Fisher Scientific) was added. The reaction was stopped by the addition of 2M HCl, and the absorbance was measured at 450 nm using a Spectra Max 190 plate reader (Molecular Devices, San Jose, USA). Alternatively, for measurement of Spike-specific IgG in culture supernatants, ELISA plates (Nunc Maxisorp, flat-bottomed, Thermo Fisher Scientific) were coated with Spike antigen at 2 μg/mL or decreasing quantities of purified IgG reference standard (Athens Research and Technology, Athens, GA, USA) starting at 200 ng/mL, overnight at 4 °C. The next day, plates were washed and blocked with 5% (*w*/*v*) non-fat milk powder in PBS-T for 1 h at room temperature. The plates were washed before the addition of antibody-containing culture supernatant samples at three-fold dilutions ranging from 1:5 to 1:1215 and incubation for 90 min at room temperature. Plates were washed before the addition of anti-IgG-HRP conjugated antibody (Southern Biotech, Birmingham, AL, USA ) and incubation for 90 min at room temperature. Plates were washed, followed by the addition of OPD substrate (Sigma-Aldrich, St. Louis, MO, USA). Plates were developed for 10 min at room temperature, resulting in a color change, and the reaction was stopped by the addition of 2M H_2_SO_4_. The absorbance was read at 490 nm using a Varioskan LUX (Thermo Scientific) with the SkanIt Software (version 7.1). Notably, the titrated quantities of IgG reference standard coated directly into duplicate wells of each ELISA plate enabled interpolation of Spike-specific IgG binding titers into IgG equivalents [14,39] using SpotStat Version 1.6.6.0 software (CTL).

### 2.5. B Cell ELISPOT Assays

ELISPOT plates were coated according to previously described methods [40]. Briefly, for enumeration of pan IgG^+^ ASCs irrespective of antigen specificity, ELISPOT plates were coated with anti-Igκ/λ capture antibody diluted in Diluent A contained in the human IgG Single-Color Enzymatic ImmunoSpot^®^ kit (from CTL) according to the manufacturer’s instructions. Alternatively, for the enumeration of SARS-CoV-2 Spike-specific IgG^+^ ASCs using the optimized affinity capture coating method [41], ELISPOT plates were first coated with purified anti-His antibody at 10 µg/mL in Diluent A (provided in CTL’s affinity coating kits) overnight at 4 °C. The rationale for affinity coating is that it enables high-density antigen coating as required for the capture of ASC-derived antibody on the membrane while reducing the quantity of antigen required. As protein-binding to the PVDF membrane occurs via hydrophobicity, both the capture antibody, or in the case of direct antigen-coating, the antigen itself, need to be hydrophobic to bind eefectively. With antigens that are weakly or non-hydrophobic, like Spike, the affinity coating method helped to enable the generation of pristine ImmunoSpot data. The following day, ELISPOT plates were washed and coated overnight at 4 °C with the His-tag labeled recombinant Spike protein at 10 µg/mL in Diluent A. After overnight incubation, ELISPOT plates were washed again and blocked with cRPMI medium for 1 h at 37 °C. The polyclonally stimulated PBMC samples were harvested from the 6-well culture plates and washed with PBS prior to counting with acridine orange and propidium iodide (AO/PI) (Biotium, Fremont, CA, USA) staining using a CellDrop^TM^ cell counter (DeNovix, Cambridge, UK). Following centrifugation, the cultured cells were resuspended in cRPMI medium at 5 × 10^6^ live cells/mL for measuring Spike-specific IgG^+^ ASCs or 2 × 10^5^ live cells/mL for measuring pan IgG^+^ ASCs. The cells were serially diluted 2-fold in round-bottom 96-well tissue culture plates (Sigma-Aldrich, St. Louis, MO, USA) and then immediately transferred into ELISPOT plates. ELISPOT plates were incubated for 4–6 h at 37 °C, 5% CO_2_. Plate-bound spot-forming units (SFUs), each representing the secretory footprint of single IgG^+^ ASCs, were visualized using the detection reagents provided in the human IgG Single-Color Enzymatic ImmunoSpot^®^ kit (from CTL) according to the manufacturer’s instructions.

### 2.6. ImmunoSpot^®^ Image Acquisition and SFU Counting

Plates were air-dried prior to scanning on an ImmunoSpot^®^ S6 Light M2 Analyzer (CTL). Pan- and Spike-specific IgG^+^ SFUs were then enumerated using ImmunoSpot^®^ Single-color Studio Software Version 1.7.35.1 and the B cell ELISPOT-specific IntelliCount™ algorithm for SFU detection [42]. Assay-specific minimal size and intensity thresholds were applied for more precise enumeration of pan- or Spike-specific IgG^+^ SFUs.

### 2.7. Flow Cytometry Staining Procedure

The antigen probe (biotinylated Spike protein) was incubated at 4 °C for 1 h with either SA-PE (1:100) or SA-APC (1:100) at a 4:1 molecular ratio of Spike:streptavidin (SA) in PBS. After thawing, PBMCs were washed with PBS. The tetrameric complex of probe-SA-PE or probe-SA-APC was incubated at 4 °C for 1 h with 10^7^ PBMCs. A decoy SA-AF647 probe was used to detect non-specific streptavidin interactions so that these events could be excluded during analysis. The cells were washed with PBS before the addition of 5 μL LIVE/DEAD^TM^ fixable dead cell stain (UV Blue, Thermo Fisher Scientific, 1:40) for 20 min and then washed with flow buffer (PBS supplemented with 2% fetal calf serum). To avoid nonspecific binding, the samples were incubated with 5 μL Human TruStain FcX™ and 5 μL True-Stain Monocyte BlockerTM (both BioLegend, San Diego, CA, USA) for 10 min.

Our flow panel was designed to be able to phenotype memory B cells (CD19^+^, CD20^+^, IgD^-^negative) while gating out T cells (CD3^+^), monocytes (CD14^+^), and NK cells (CD56^+^). All anti-human monoclonal antibodies were purchased pre-conjugated (see Table S1) and were titrated for optimum staining performance. Following the blocking step, the antibody cocktail- was incubated with the cells for 30 min at room temperature in the dark. Following washing, fixing was performed using 500 μL of 1% PFA (Biolegend) for 20 min. Cells were washed with flow buffer, and the pellet was resuspended in 300 mL of flow buffer. Samples were acquired on a 5-laser Aurora spectral flow cytometer (Cytek, Fremont, CA, USA).

### 2.8. Statistical Methods

Statistically significant differences between the pre-COVID-19, convalescent, and 2024 PBMC cohorts in the ELISA, ELISPOT, or probe-based flow cytometry assays were determined using a one-way ANOVA (with Tukey’s post hoc test). Pearson correlation analysis and regressions were also performed, and the *R*^2^ values and *p*-values are shown in the corresponding figures. Each of these statistical analyses was performed using version 10.4.0 of GraphPad Software (La Jolla, CA, USA). Correlation of values, denoted as *R*^2^, for SFU frequency measurements based on three or more cell inputs from the dilution series were calculated using the ImmunoSpot^®^ Single-color Studio software (Version 1.7.35.1).

## 3. Results

### 3.1. Rationale and Study Design

Reference PBMCs, comprising negative and positive controls, is critical for assay development and the comparison of test systems. In particular, the availability of negative controls is a challenge for immune monitoring, as antigens to which humans have verifiably not been exposed are rare. Therefore, the emergence of SARS-CoV-2 and its appearance in the USA in January 2020 [37] provided a unique test system for immune assay comparisons. PBMCs isolated and cryopreserved in the USA before November 2019 originate from donors who are unequivocally naïve to this virus and serve as perfect negative controls for measuring immune responses targeting SARS-CoV-2 antigens. Therefore, one of the cohorts included in our studies constituted 12 subjects’ PBMCs that were cryopreserved before the spread of this virus and designated the “pre-COVID-19” cohort.

**Figure 2:**
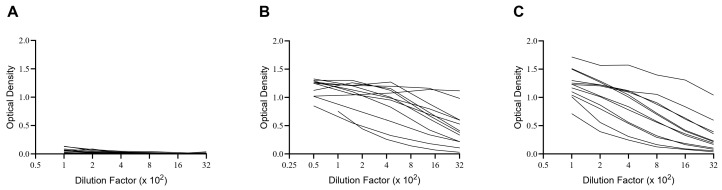
Spike-specific antibody curves generated by serial dilution of plasma from the three cohorts studied. (**A**) Pre-COVID-19 donors, i.e., subjects whose blood was drawn before November 2019. (**B**) The convalescent cohort, with blood drawn in 2020, 3–6 months after PCR-verified SARS-CoV-2 infection of the first wave. (**C**) The 2024 cohort, consisting of random individuals, bled during that year, by which time SARS-CoV-2 had become endemic. The demographic of these subjects is defined in Table 1. The serum of each subject was tested at the dilutions specified on the *x*-axis, with the corresponding OD reading shown on the *y*-axis. The ELISA was performed as described in Section 2.

Moreover, the initial intense efforts to identify emerging SARS-CoV-2 infections also provided a unique opportunity for immune monitoring. Namely, PCR-positive donors with clinical symptoms of COVID-19 were verifiably exposed to SARS-CoV-2 and hence serve as the ideal positive control group for detecting an immune response directed against SARS-CoV-2 antigens. PBMCs collected from 12 subjects 3–6 months after PCR-verified SARS-CoV-2 exposure during the first wave of infections thus constituted our positive controls, the “convalescent” cohort. Finally, we tested PBMCs collected from 12 subjects in 2024 with unknown histories of SARS-CoV-2 antigen exposure (our 2024 cohort). By this time, however, most subjects can be expected to have either been previously infected and/or vaccinated—in either case, developing memory B cells (B_mem_) and antibodies to the SARS-CoV-2 Spike protein. The results generated when testing plasma from these subjects for Spike-specific IgG are shown in Figure 2. Notably, while none of the pre-COVID-19 donors possessed elevated levels of Spike-specific IgG, all subjects in the other two cohorts did, albeit at highly variable levels. Did the latter individuals also develop detectable numbers of Spike-specific IgG^+^ B_mem_, and if so, which of the three test systems for monitoring B_mem_ is best suited to reliably detect and quantify them?

The PBMCs were cryopreserved following a protocol that permits recovery of fully functional B_mem_ after the cells are thawed [27,36]. This was verified for all PBMCs tested: data showing the post-thaw viability and pan (total) IgG^+^ ASC activity irrespective of antigen specificity following polyclonal stimulation are provided in Figure S1A and S1B, respectively. As no significant differences in either of these parameters were seen among the PBMCs in the three cohorts, we conclude that they all had been cryopreserved to expectations, and therefore any difference in Spike-specific B_mem_ detection in individual PBMC samples is intrinsic to that donor, rather than a consequence of variable success in PBMC cryopreservation.

The design of our B_mem_-detection testing strategy, which was carried out blinded to the laboratory experimenter performing the tests at Northumbria University, is shown in Figure 1. Specifically, two vials of PBMCs from each donor were thawed, from which 10 × 10^6^ PBMCs were allocated for Spike probe staining, since this technique requires more cells compared to the other two techniques [27,34,43]. Additionally, 5 × 10^6^ or 4 × 10^6^ PBMCs were subjected to polyclonal stimulation to transition resting B_mem_ into antibody-secreting cells (ASCs) for testing in ImmunoSpot assays or generation of antibody-conditioned culture supernatants, respectively.

### 3.2. Comparison of Sensitivity, Specificity, and False Negative Results Using Three Different Techniques for B_mem_ Detection

**Figure 3:**
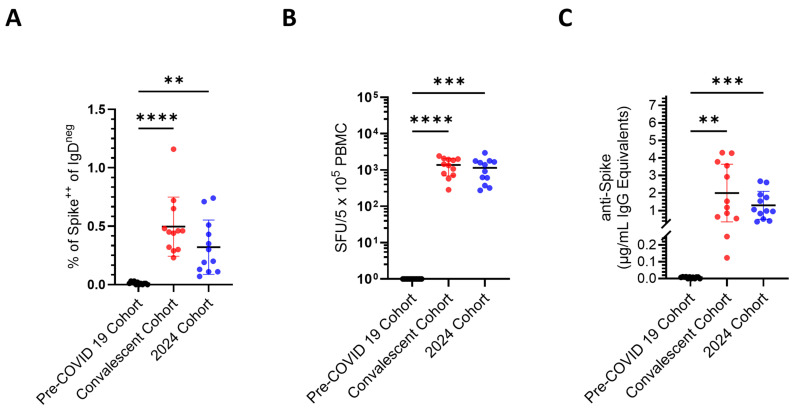
Spike antigen-specific IgG^+^ B_mem_ frequency measurements in the specified cohorts. PBMCs were obtained from three defined cohorts: pre-COVID-19 samples collected prior to the pandemic (*n* = 12) in black, PCR-confirmed SARS-CoV-2 convalescent donors (*n* = 12) in red, and donors collected in 2024 (*n* = 12) in blue. (**A**) Frequency of class-switched (IgD^neg^) Spike-specific B cells was determined by probe-based flow cytometry (see Figure 4 for gating strategy). (**B**) The number of Spike-specific IgG^+^ ASCs per 5 × 10^5^ PBMCs as measured by B cell ImmunoSpot. (**C**) Concentration of Spike-specific IgG (µg/mL) in culture supernatants following 11 days of B-Poly-S stimulation, assessed by an ELISA. Results for the individual donors are represented by a dot each, with mean ± SD shown for each cohort. Statistical significance was determined by one-way ANOVA with Tukey’s post hoc test (** *p* < 0.01, *** *p* < 0.001, **** *p* < 0.0001).

Specificity is defined in clinical diagnostics as the proportion of negative test results out of all truly negative samples. High specificity will identify all samples without the condition of interest (in this case, generation of B_mem_ specific for the SARS-CoV-2 Spike), that is, excluding false positive results in pre-COVID-19 samples. As seen in Figure 3A–C, this criterion was met for all three B_mem_ detection systems. It should be noted, however, that to achieve diagnostic sensitivity in flow cytometry, a negative cutoff of 0.03% Spike^+^ CD19^+^ IgD^neg^ events was required for the pre-COVID-19 PBMC samples (Figure S2A). Consequently, this detection system required a higher cut-off value to be established to achieve diagnostic specificity, and this could obscure detection of low frequencies of antigen-specific B_mem_. In contrast, testing of pre-COVID-19 samples in ImmunoSpot yielded no detectable spot-forming units (SFU), that is, secretory footprints originating from individual Spike-specific IgG^+^ B_mem_-derived ASCs, even when 5 × 10^5^ PBMCs were tested per well (Figure S2B).

**Figure 4:**
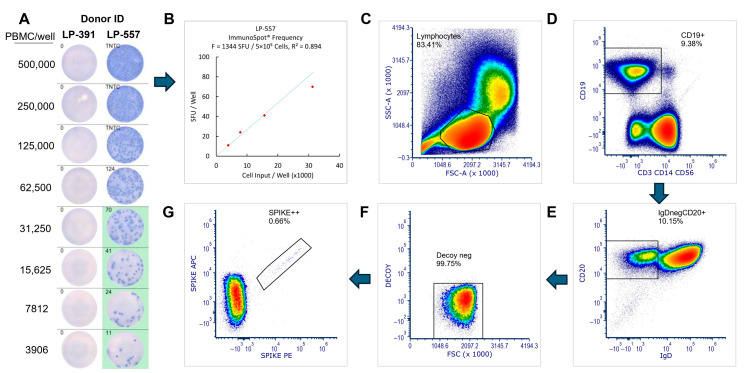
Representative data obtained by ImmunoSpot^®^ and by probe staining. (**A**) Raw data for a Spike antigen-specific IgG-detecting ImmunoSpot^®^ assay are shown, in which the PBMC of a pre-COVID-19 donor (LP-391, on the left) and a convalescent donor (LP-557, on the right) were plated in the specified serially diluted cell numbers. The secretory footprints (Spot Forming Units, SFUs) as counted automatically for each well by the ImmunoSpot^®^ software are shown in the upper right corner of each well image. As crowding and ELISA effects interfere with precise counting of SFUs at high SFU numbers, the wells in which the PBMC numbers plated per well and SFUs counted relate linearly to each other are automatically identified by the software and are highlighted by a green field. From the linear (in this case, 4) data points, the software automatically extrapolates the frequency, as shown in Panel (**B**), R^2^ = 0.894. Panels (**C**–**G**) show the successive gating for the probe staining of the same convalescent donor’s PBMC. Colors are heatmaps for cell numbers with red being the densest. Eventual Spike double-positive cells, G, are expressed as % of cells from F.

Sensitivity in clinical diagnostics measures the proportion of positive test results out of all truly positive samples. In our case, each B_mem_ test’s sensitivity was its ability to correctly identify PBMCs of donors who had verifiably been exposed to Spike in the convalescent cohort (the true positives), while minimizing the number of false negative results in this cohort. Overall, a 100% sensitivity criterion was met by flow cytometry and by ImmunoSpot for all 12 donors in the convalescent cohort (Figure 3A,B) and also by ELISA when measuring antibody-conditioned supernatants (Figure 3C). Representative data from ImmunoSpot and antigen probe-based flow cytometry assays, both of which provide single-cell resolution, are shown in Figure 4. In all three B_mem_ assays, however (similar to plasma antibody levels detected by ELISA), considerable inter-individual variations in positivity were seen, to be dissected in the following.

### 3.3. Inter-B_mem_ Assay Correlations

**Figure 5:**
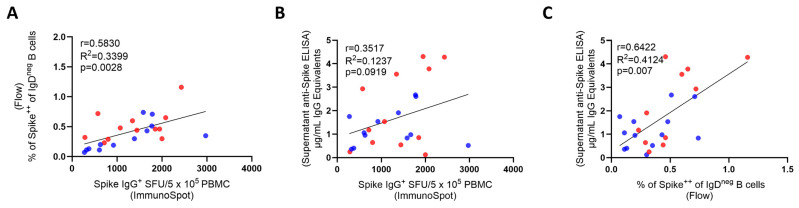
Correlations for the data obtained in the specified B_mem_ detection assays. (**A**) ImmunoSpot vs. probe staining: significant r = 0.5830, *p* = 0.0028, R^2^ = 0.3399; (**B**) ImmunoSpot vs. supernatant r = 0.3517, *p* = 0.0919, R^2^ = 0.1237 trend but not significant; and (**C**) supernatant vs. probe staining: significant r = 0.6422, *p* = 0.007, R^2^ = 0.4124. Each dot represents a PBMC sample of the Convalescent (in red) and 2024 Cohorts (in blue) combined. Pre-COVID-19 samples, all being negative in all assays, have been excluded from the correlation analysis. Pearson correlation analyses were performed; r, R^2,^ and *p* values are shown in each panel. All three assays demonstrated concordant detection of Spike-specific memory B cell responses from a common starting material, but with B being only a trend (*p* = 0.0919).

Both antigen probe staining and ImmunoSpot detect individual Spike-specific B_mem_. However, the two test systems rely on different approaches: in the first, PBMCs are stained ex vivo without any prior treatment or culture. For ImmunoSpot, the PBMCs first need to be polyclonally stimulated for 5 days to convert resting B_mem_ (that do not actively secrete antibody) into ASCs whose secretory footprints can be detected. Presently, we can only speculate what proportion of B_mem_ transitions into ASCs during the culture. For the detection of specific antibody reactivity in supernatants from polyclonally stimulated PBMCs, a culture duration of 11 days was found to be optimal [28]. While the magnitude of the positive signal seen within each of these readouts can be expected to depend on the frequency of antigen-specific B_mem_ present in each test sample, to our knowledge, the extent to which the results provided by each assay qualitatively correlate with each other has not been established. Figure 5A–C show that the test results obtained using the three approaches correlated with each other (as the pre-COVID-19 samples were all negative, only the convalescent and the 2024 sample cohorts were included in this analysis). While the correlation of the results is not particularly high (as one might expect based on the different means by which B_mem_ were measured), given that probe-based flow cytometry assesses the B_mem_ cells directly ex vivo, while the other two require polyclonal stimulation for 5 days for B cell ELISPOT and 11 days for testing of antibody titers in an ELISA assay, it is expected that there will be some variability in the relationships of the three assays when compared side-by-side. Nevertheless, all three assays were able to determine exposure to Spike. Moreover, all three assays were successful in identifying donors with high, low, and intermediate Spike-specific B_mem_ frequencies.

## 4. Discussion

A strength of this study is that it leveraged a clearly defined immune monitoring scenario in which naivety or exposure to an antigen could be unambiguously verified. There are few antigenic systems for humans to which this scenario applies. Unlike SARS-CoV-2 exposure before the emergence of this virus (and in the meantime, no longer even for SARS-CoV-2), most viral infections are endemic to the human population, occur early on in life, and often go clinically undiagnosed. While seropositivity is presently the gold standard in the clinical diagnostic arena for verifying past infections with such viruses, sero-diagnostics can also provide false negative data, whereas the presence of antigen-specific B_mem_ and T cells can reveal prior antigen exposure and the development of immunological memory in seronegative individuals [13,14,44,45,46]. Establishing a naïve vs. primed immune state becomes challenging, also with autoantigens, including tumor-associated antigens.

One weakness/limitation of this study is that it relied on positive control samples obtained from convalescent donors (with PCR-verified SARS-CoV-2 infections) in which the antigen exposure was relatively recent. Consequently, the frequencies of Spike-specific B_mem_ in these test subjects were substantially higher than typically measured for other viral antigens (Table S2). Additionally, specific details of the 2024 cohort’s prior COVID-19 vaccination and/or SARS-CoV-2 infection history were not available and may have influenced the magnitude of Spike-specific antibody titers and/or B_mem_ frequencies detected in these samples. Nevertheless, our results demonstrate that all three B_mem_-detecting test systems enabled a quantitative (or at least semi-quantitative) assessment of B_mem_ when the frequency of these cells was in the higher range; at this point it remains an open question which of these assay approaches will be best suited for immune diagnostics when antigen-specific B_mem_ are present only in the low (and very low) frequency range. To this end, our group recently demonstrated that low frequencies of SARS-CoV-2 NCAP-specific B_mem_-derived IgG^+^ ASCs were reliably detected by ImmunoSpot in post-COVID era PBMC samples by extending the limit of detection (see Table S3, Kirchenbaum et al., manuscript in preparation). Furthermore, in a prior study published by Dan et al. [47], NCAP probe-binding B_mem_ were not uniformly detected in donors that recently recovered from SARS-CoV-2 infection.

When selecting a B_mem_-detecting assay for immune monitoring, additional emphasis may also be placed on the clinically useful information it can provide. For instance, antigen probe staining has the advantage of enabling in-depth phenotypic analysis and positive sorting of antigen probe-binding B cells for downstream sequencing/cloning of B cell receptors, which may be crucial in research or some clinical settings. However, this technique has the drawback of an increased cost, technical effort, and a requirement for a large number of PBMCs (10^7^ in this study). Moreover, the immune diagnostic utility of surface phenotyping and genetic repertoire analysis of antigen-specific B cells remains unclear. Furthermore, the high cost of suitable multi-parametric flow cytometry instrumentation, the multitude of staining reagents required, and the personnel expertise required to generate such data also should be considered.

As the only cell type capable of secreting antibodies (immunoglobulins) in the body are B cells, and since IgG is only secreted by B_mem_ that previously underwent T-helper cell-dependent Ig class switching in vivo, the quantitative (ImmunoSpot) or semi-quantitative (supernatant) measurement of antigen-specific IgG-producing cells provides information on the number of B_mem_ and their functionality in PBMCs without the need for additional phenotypic verification. Furthermore, the simplicity of the ImmunoSpot test procedure, the substantially lower cost of instrumentation and reagents needed, the economic PBMC utilization, and the fully automated image analysis of the assay results, are additional arguments favoring this methodology.

## 5. Conclusions

Immune monitoring of B_mem_ is an evolving field and will inevitably improve our understanding of B-cell-mediated immune reactions that govern health and disease. The suitability of the different B_mem_-detecting test systems, their ease of implementation, and the diagnostically relevant information they provide will ultimately influence the preferred method(s) utilized. Each assay was able to discern Spike-exposed individuals from naïve ones, with ImmunoSpot suggesting superior sensitivity and specificity. ImmunoSpot and flow cytometry results were closely correlated. Depending on the rationale of a given proposed study, all three assays can be used together or individually to inform on B cell memory in antigen-primed individuals when such B_mem_ occur in the mid- to high-frequency range; and they all broadly concur.

## Figures and Tables

**Table 1:** Cohort demographics (SD standard deviation).

Cohort	Pre-COVID-19 Donors	Convalescent Donors	2024 Donors
n	12	12	12
Age	36.58 (SD 10.93)	47.58 (SD 9.376)	41.33 (SD 7.075)
Collection date (start to end)	Apr-2017 to Oct-2019	Jul-2020 to Dec-2020	Feb-2024 to June-2024

## Data Availability

The data generated in this study will be made available by the authors, without undue reservation, to any qualified researcher.

## Supplementary Materials

The following supporting information can be downloaded at: https://www.mdpi.com/article/10.3390/vaccines14010020/s1, Figure S1: PBMC viability upon thawing, and pan IgG+ ASC activity following 5-days of in vitro polyclonal stimulation.; Figure S2: Representative flow cytometry and ImmunoSpot® results for pre-COVID 19 cohort donors.; Table S1: Reagents included in the probe-based flow cytometry surface phenotyping panel; Table S2: Variable donor reactivity for SARS-CoV-2 and other antigens; Table S3: Extending the limit of detection for measuring SARS-CoV-2 antigen-specific Bmem-derived IgG+ ASC.

## Abbreviations

B_mem_	memory B cell(s)
PBMCs	peripheral blood mononuclear cells
ASC	antibody-secreting cells
