# Supplementary material for: A Comparison of Flow Cytometry-based versus ImmunoSpot- or Supernatant-based Detection of SARS-CoV-2 Spike-specific Memory B Cells in Peripheral Blood
Source: Vaccines (Basel). 2025 Dec 24;14(1):20. doi: 10.3390/vaccines14010020 (PMC12846400; doi:10.3390/vaccines14010020)
Supplement: Supplementary file 1 [file vaccines-14-00020-s001.zip › vaccines-4024453-supplementary.pdf]

Stylianou et al., Supplementary Materials

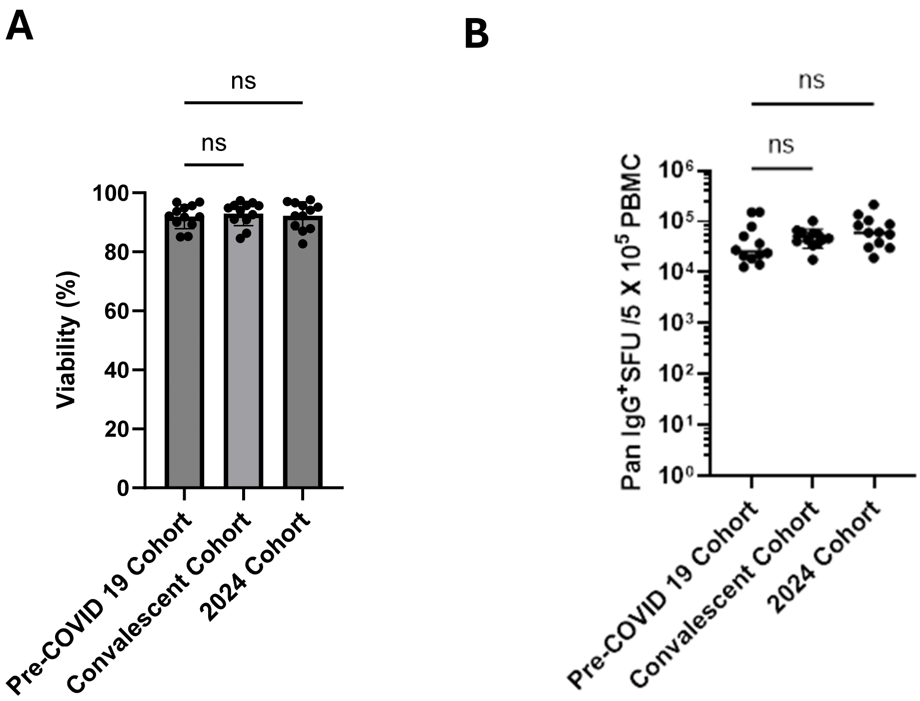

Supplementary Figure S1: PBMC viability upon thawing, and pan IgG+ ASC activity following 5-days of in vitro polyclonal stimulation. Data represent results from n=36 donors, comprising three cohorts of n=12 donors each. Panel A depicts the viability of donor PBMC upon thawing of the cryopreserved peripheral blood mononuclear cells (PBMC). Notably all donors had >90% live cells post thawing assuring good cell recovery. Five million cells were subjected to a 5-day polyclonal stimulation. Panel B depicts the frequencies of pan IgG+ antibody-secreting cell (ASC) generated spot-forming units (SFUs) per 5 x 10^5 PBMC cell input. Notably, there was not a statistically significant difference between the three cohorts in cell viability following thawing or pan IgG+ ASC frequency following polyclonal stimulation.

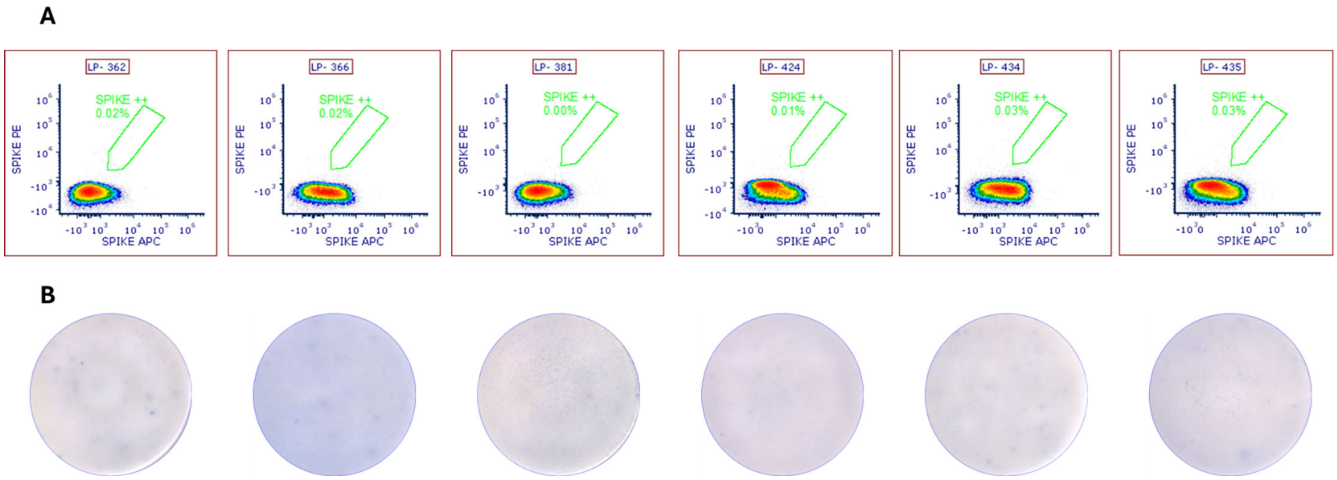

Supplementary Figure S2: Representative flow cytometry and ImmunoSpot® results for pre-COVID 19 cohort donors. Panel A shows the percentage of Spike^{++} IgD^{neg} B cells identified by probe-based flow cytometry post the subsequent gating from Singlet cells to Live cells, lymphocytes, CD19 positive events, IgD negative events, SA-AF 647 negative (negative for non-specific binding to the empty biotinylated SA-AF647) and lastly, gating on the double positive (++) events for SPIKE-PE and SPIKE APC. Notably in the pre-COVID 19 era samples there were very low frequencies of positive events at the terminal gating of SPIKE APC/PE events. Panel B shows the corresponding Spike-coated ImmunoSpot® wells inputed with 5 x 10^5 PBMC from the same donors following polyclonal stimulation. Note the complete lack of SFU in B, but presence of a low level of background noise in A.

Specificity	Clone Name	Fluorochrome	Supplier
CD3	HIT3a	PerCP	BioLegend
CD14	HCD14	PerCP	BioLegend
CD56	5.1H11	PerCP	BioLegend
IgD	IA6-2	BV480	BD Biosciences
CD20	2H7	Spark Violet™ 538	BioLegend
CD19	HIB19	BYG710	Cytek
SA	-	AF647	BioLegend
SA	-	APC	BioLegend
SA	-	PE	BioLegend
Viability UV	-	UV Blue	BioLegend

Supplementary Table S1: Reagents included in the probe-based flow cytometry surface phenotyping panel

Donors	SARS-CoV-2 S-antigen	SARS-CoV-2 NCAP	CA/09 (A/H1)	TX/12 (A/H3)	Phuket/13 (B/Yam)	TTHc (Tetanus)	EBNA1 (EBV)	gH Pentamer (HCMV)	6xHis
LP725	3	2	13	9	7	2	8	3	0
LP726	>100	13	93	13	21	3	21	71	0
LP727	>100	>100	83	38	41	1	0	2	0
LP728	67	6	14	8	5	1	2	3	0
LP730	41	51	67	15	40	8	14	43	0
LP731	>100	20	>100	48	>100	1	11	1	0
LP735	>100	66	5	4	7	1	4	9	0
LP736	77	21	11	6	9	1	11	2	0
LP738	>100	5	3	23	7	2	13	12	0
LP739	>100	>100	36	46	28	9	>100	0	1
LP740	>100	>100	29	47	40	10	79	27	2
LP741	26	21	>100	21	38	16	84	>100	2
LP749	72	18	11	8	5	8	29	2	2
LP751	>100	>100	38	39	60	66	67	16	0
LP756	>100	36	34	17	16	4	28	2	4
LP757	>100	8	>100	23	87	5	22	0	0
LP758	>100	>100	>100	>100	39	15	36	0	0
LP760	>100	30	>100	69	73	5	27	>100	2
LP761	>100	36	>100	86	83	6	>100	6	1
LP769	>100	>100	>100	58	36	11	1	1	1

Supplementary Table S2: Variable donor reactivity for SARS-CoV-2 and other antigens. Cryopreserved PBMC collected from post-COVID era donors were evaluated in ImmunoSpot® following polyclonal stimulation for IgG^{+} ASC reactivity against the SARS-CoV-2 Spike (S-antigen) and Nucleocapsid (NCAP) proteins, along with commonly encountered antigens representing influenza vaccine strains (CA/09, TX/12, or Phuket/13), Tetanus (TTHc), EBV (EBNA1) or HCMV (gH Pentamer). Wells coated with an anti-His antibody followed by addition of 6xHis peptide served as the negative control. Individual donors' responses are denoted as the number of spot-forming units (SFUs) per 3×10^5 PBMC, with ">100" indicating responses that were above the upper limit of quantification of the assay as performed. The data are reproduced from Becza et al, Vaccines 2025, 13 (7), 765.

		S-antigen[a]	NCAP[a]	6xHis[a]	BSA[a]	Pan IgG[b]
Pre-COVID	LP366	0	0	1	0	39,800*
	LP377	3	6	3	0	157,800*
	LP381	0	0	0	0	19,933*
	LP391	0	9	0	0	57,733*
	LP413	3	4	0	0	299,600*
	LP424	1	2	0	0	49,467*
	LP426	0	1	2	0	257,200*
	LP432	2	4	1	1	70,200*
	LP434	0	1	3	1	133,067*
	LP475	0	5	4	1	250,000*

Pan IgG $\bar{x} \pm \sigma$
133,480 ± 103,032

		S-antigen[b]	NCAP[a]	6xHis[a]	BSA[a]	Pan IgG[b]
Post-COVID	LP695	967*	35	3	1	156,400*
	LP696	5067*	49	1	0	60,900*
	LP728	210	35	2	0	202,133*
	LP741	170	21	0	0	229,867*
	LP757	5200*	31	1	0	114,133*
	LP815	1880*	24	0	0	63,900*
	LP820	900*	44	0	0	66,100*
	LP827	730*	31	0	0	124,600*
	LP828	4960*	21	0	0	52,400*
	LP836	1680*	25	0	0	86,800*

Pan IgG $\bar{x} \pm \sigma$
115,723 ± 62,567

ns

[a] Spot-forming units (SFUs) were aggregated from 10 replicate wells seeded with 2 x 10^5 PBMC

[b] SFU values were extrapolated to 2 x 10^6 PBMC

*SFU value determined through serial dilution of donor PBMC

Supplementary Table S3: Extending the limit of detection for measuring SARS-CoV-2 antigen-specific B_{mem}-derived IgG^+ ASC. Cryopreserved PBMC collected from pre-COVID (n=10) and post-COVID (n=10) era donors were evaluated in ImmunoSpot® following polyclonal stimulation for B_{mem}-derived IgG^+ ASC reactivity against the SARS-CoV-2 Spike (S-antigen) and Nucleocapsid (NCAP) proteins, along with negative controls (6xHis peptide and BSA) in an attempt to detect rare IgG^+ B_{mem} present in pre-COVID donors through extending the limit of detection to 1 in 2 x 10^6 PBMC. The pan IgG values serve as a positive control and verified successful polyclonal stimulation of PBMC and their transition of resting B_{mem} into ASCs. The mean ± SD of pan IgG^+ ASC in the two cohorts is shown to the right of the Pan IgG column and was not statistically different. The data are reproduced from Kirchenbaum et al, Vaccines (manuscript in progress)
